# Facial Swelling as a Primary Manifestation of Multiple Myeloma

**DOI:** 10.1155/2015/319231

**Published:** 2015-07-01

**Authors:** Anju E. Thomas, Seema Kurup, Renju Jose, Cristalle Soman

**Affiliations:** Department of Oral Medicine and Radiology, Amrita School of Dentistry, AIMS Campus, Ponekkara, Kochi 682041, India

## Abstract

Facial swellings are commonly encountered in the dental office, the cause of which could range from a congenital etiology to an acquired one or it may even be a manifestation of an underlying systemic disease. The clinician must have a thorough knowledge of the various clinical and imaging manifestations and the sites of occurrence of the various conditions to arrive at the appropriate diagnosis. Facial swellings can be classified into different groups which include acute swellings with inflammation, nonprogressive swellings, and slowly or rapidly progressive swellings. The various imaging modalities like CT and MRI are useful for assessing the extent of the swelling as well as evaluating the soft tissue and osseous involvement of the swelling. Multiple myeloma represents clonal proliferation of plasma cells and is a condition in which a facial swelling might be present, though not common. This paper reports a case of a patient with a unilateral facial swelling, which on investigation led to a diagnosis of multiple myeloma.

## 1. Introduction

Facial swellings are commonly encountered in the dental office and can be a cause of worry to both the patient and the dentist. They may arise due to a wide range of causes ranging from a congenital etiology to an acquired one [1]. A detailed record of the clinical history and physical manifestations are considered as important factors in the evaluation of facial swelling. Recent advances in the field of imaging have enabled the clinician to determine the presence and extent of disease which will also aid in treatment planning. The clinical manifestations of facial swellings may be categorized into four groups: acute swellings with inflammation and nonprogressive, rapidly progressive, and slowly progressive swellings. Acute swellings are seen in lymphadenitis, odontogenic infections, and abscesses. Nonprogressive swellings are suggestive of a congenital anomaly whereas slowly progressive swellings are seen in vascular malformations, haemangioma, and fibrous dysplasia. Rapidly progressive swellings are usually associated with malignancies. This paper reports a case of facial swelling which proved to be the primary manifestation of multiple myeloma.

## 2. Case History

A 58-year-old female patient presented with a swelling on the left side of face which had evolved over the previous two months. Although the patient was treated with antibiotics, the swelling continued to enlarge to reach its present size. The patient had neither dryness of the mouth nor increased salivation, and the swelling was persistent. The patient did not have any associated fever or paresthesia. She was a diagnosed diabetic patient on oral hypoglycemics for the past 4 years.

**Figure 1:**
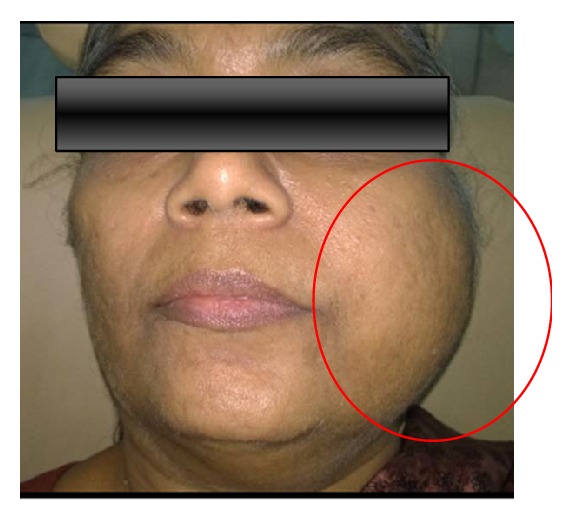
A diffuse extraoral swelling in the middle and lower one-third of the face.

On extraoral examination, a diffuse ovoid swelling was seen on the left middle and lower third of the face extending anteroposteriorly from the nasolabial fold to the tragus of the ear and superoinferiorly from the zygomatic arch to two centimeters beneath the lower border of the mandible. Palpation revealed a nontender, noncompressible, and nonfluctuant swelling which was firm to hard in consistency. There was a slight reduction in mouth opening (Figure 1) and the cervical lymph nodes were nonpalpable.

**Figure 2:**
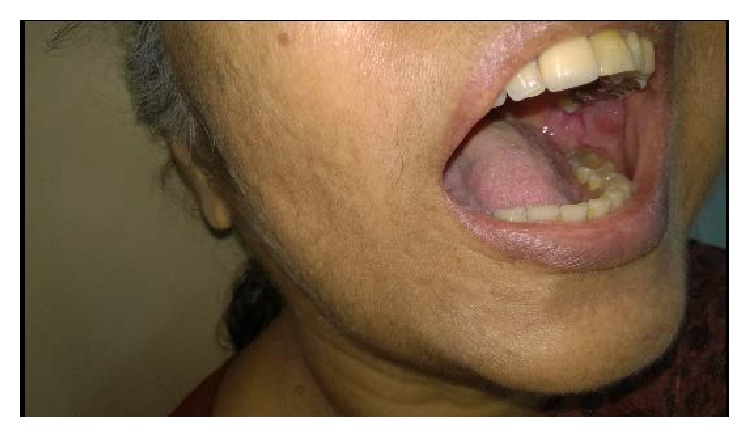
Intraoral examination reveals a normal mucosa and dentition.

Intraoral examination revealed a fixed prosthesis in relation to the upper left posterior teeth. The skin overlying the swelling and the oral mucosa were unaltered and were of normal color (Figure 2). The clinically considered diagnosis included Sjogren's syndrome or a parotid gland tumor.

Various hematological tests included routine blood tests and an ANA screen (Table 1) to rule out autoimmune conditions like Sjogren's syndrome.

**Figure 3:**
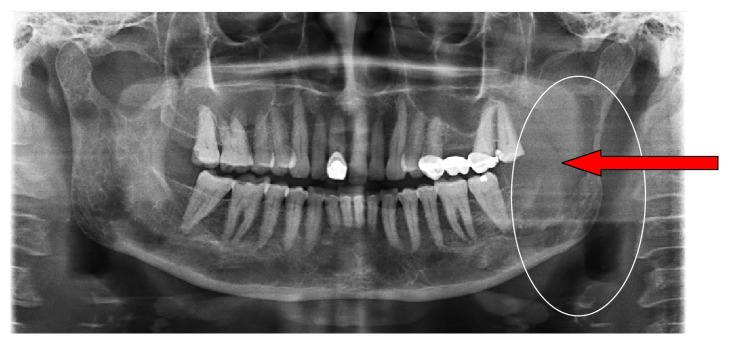
A radiolucent lesion with ill-defined ragged borders involving the left ramus, body, and coronoid process of the mandible. The right side of the mandible shows multiple punched out radiolucencies in the body, ramus, and condylar region with altered trabecular pattern.

An orthopantomograph (OPG) revealed an extensive osteolytic lesion with ill-defined margins involving the left mandibular ramus, body, and coronoid with multiple radiolucent lesions and altered trabecular patterns in the right mandibular ramus, body, and condylar region (Figure 3). Ultrasonography revealed a hypoechoic solid lesion anterior to the superficial lobe of left parotid. FNAC revealed smears with low cellularity and aggregates of binucleated and multinucleated plasma cells.

**Figure 4:**
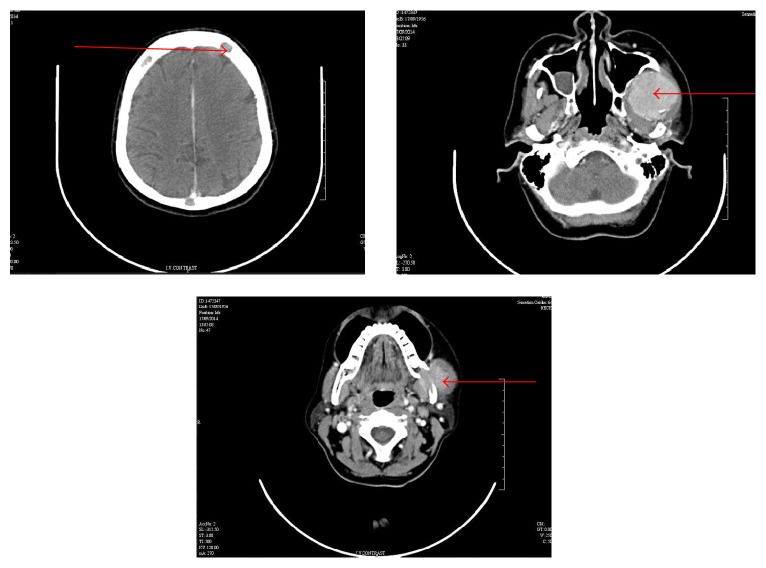
Axial CT section: Lytic lesion in left frontal bone and an expansile lytic and destructive lesion in the left ramus of the mandible involving the left infratemporal fossa and masseteric space and indenting the lateral wall of the left maxillary sinus.

**Figure 5:**
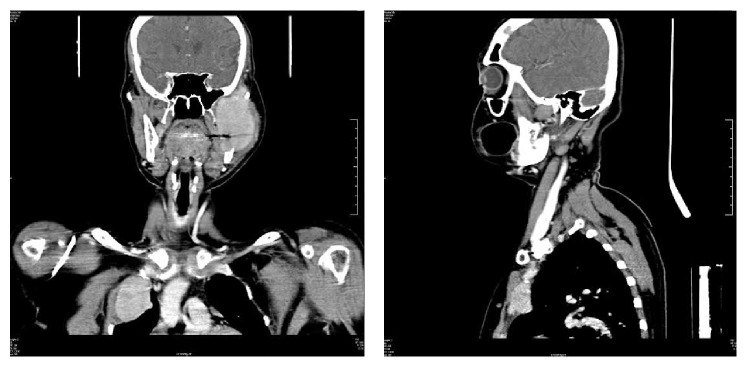
Coronal and sagittal sections of CT demonstrating the extent of the lesion.

Contrast enhanced CT showed an expansile lytic and destructive lesion in the left ramus of the mandible with associated soft tissue component and a few enlarged lymph nodes in level 3 on the left side. A lytic lesion was noted in the left frontal bone as well as the right costochondral joint of the second rib (Figures 4 and 5). The radiological differential diagnoses considered were metastatic carcinoma or multiple myeloma taking into account the age of the patient and the ragged margin of the lesion.

Based on the extensive skeletal involvement and the FNAC report, a bone marrow biopsy was done to assess if the patient had multiple myeloma. The aspirate revealed a cellular marrow with hypocellular fragments and poor cellular characteristics and 18% of plasma cells of which few were binucleate. This warranted further confirmatory investigations for multiple myeloma. Biochemical tests for serum calcium and serum creatinine were within normal limits. Serum electrophoresis revealed decreased albumin (3.98 g/dL), increased globulin (4.8 g/dL), and increased beta-2 microglobulin (2.8 g/dL) levels (Table 2). The immunoglobulins in the serum were assessed quantitatively and IgA (3000 mg/dL) was found to be raised and the kappa lambda ratio was found to be 1 : 5 (Table 3).

A final diagnosis of multiple myeloma was made based on the clinical presentations and investigations, which satisfied the revised diagnostic criteria of International Myeloma Working Group (IMWG) [2].

**Figure 6:**
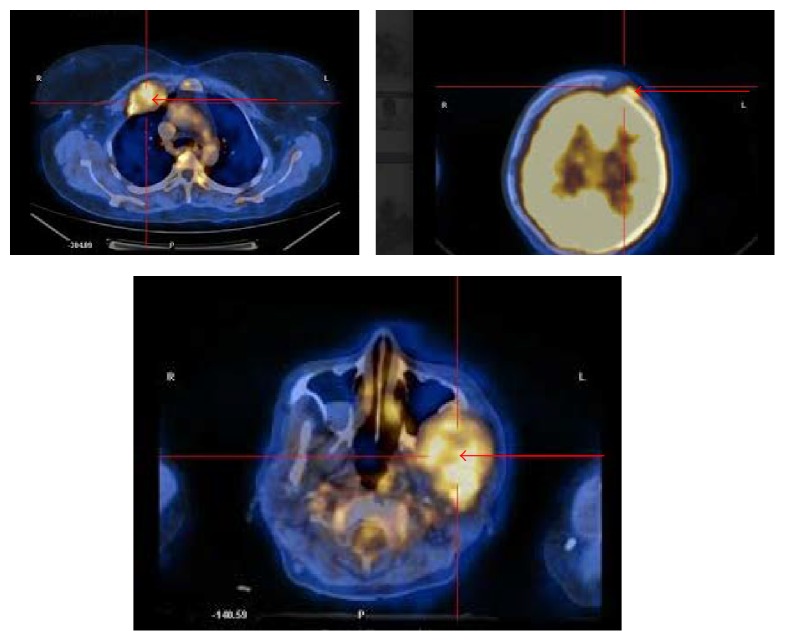
PET CT images showing extensive FDG uptake in frontal bone, maxilla, and right costochondral junction of second rib.

PET CT (Figure 6) was further done to assess the skeletal involvement and this revealed involvement of calvarium, left mandible, and right costochondral joint of second rib. The patient was subjected to both radiotherapy (3000 cGy in 12 fractions over a period of 15 days for the mandibular and parasternal lesion) and chemotherapy with CyBorD (cyclophosphamide, bortezomib, and dexamethasone).

Following 16 weeks of chemotherapy, the patient's IgA levels normalized and she is currently scheduled for autologous stem cell transplantation.

## 3. Discussion

The term “multiple myeloma” was introduced by J. Von Rustizky in 1873 when he found eight separate tumors of the bone marrow which he designated as “multiple myeloma.” The first well-documented case of multiple myeloma was reported by Samuel Solley in 1844 [3].

Multiple myeloma is a malignant disease characterized by multifocal proliferation of atypical plasma cells and by the presence in the serum of monoclonal gamma globulins, often referred to as “M” or myeloma proteins [4, 5]. The disease has a male predilection, with the average age at diagnosis being approximately 60 years [6, 7].

Multiple myeloma involves destructive lesions affecting the bone and the patients present with persistent pain in the bone, especially in the affected areas with a history of recurrent infection, fever, fatigue, and multisystem involvement [8, 9].

In our case, the patient was a fifty-eight-year-old female with manifestations in the maxillofacial region. Primary manifestation in the maxillofacial region, though not common, usually manifests as pain, swelling, paresthesia, mobility of tooth, hemorrhage, and pathological fractures [10, 11]. Bruce and Royer [12] have reported a prevalence rate of 28.8% (17 of 59 total cases) in their study while Epstein et al. [13] reported that 14.1% of 783 multiple myeloma cases had oral manifestations. Studies conducted by Lambertenghi-Deliliers et al. showed mandibular jaw involvement in all the 193 cases examined [14].

In the present case, the patient had a swelling on the left side of face which was not associated with pain, paresthesia, or mobility or displacement of the teeth.

Radiographic examinations usually reveal osteolytic lesions with irregular, noncorticated margins and multiple punched out radiolucencies with altered trabecular pattern which was seen in the present case too [15]. The osteolytic lesions have a predilection for the mandible due to the lower amount of hemopoietic marrow in the mandible and are attributed to a lymphokine called “osteoclast activating factor” [16]. Witt et al. reported that 15.6% of patients diagnosed with multiple myeloma had osteolytic lesions in mandible which were asymptomatic and 46.7% had skull manifestations [17].

The diagnosis of multiple myeloma in our case was established by a multidisciplinary diagnostic protocol involving routine investigations of both serum and urine followed by quantification of immunoglobulins. This was accompanied by cytological examination of bone marrow aspirate and a detailed radiological survey in the form of contrast enhanced CT, PET CT to assess the extent of skeletal involvement. Shah et al. have emphasized the importance of 18F-FDG PET/CT in myeloma patients as it enables detection of unsuspected sites of bone involvement, which upstages the disease [18]. The use of PET in the present case confirmed the extent of bone involvement and helped in the staging of the disease.

The patient was staged as stage 1 as per the International Staging System (ISS) (Table 4) which has a fair prognosis with a median survival time of 6–8 years [19].

Radiotherapy for the symptomatic lesion followed by chemotherapy is the treatment of choice [20]. The chemotherapeutic agents used in the treatment of multiple myeloma include melphalan, vincristine, cyclophosphamide, doxorubicin, and bendamustine in combination with corticosteroids, immunomodulating agents, or proteasome inhibitors [21]. Radiotherapy followed by a combination of cyclophosphamide, bortezomib, and dexamethasone was used in our case for 16 weeks, after which the immunoglobulin levels normalized.

## 4. Conclusion

The present case reports maxillofacial swelling as the primary manifestation of multiple myeloma, reinforcing the role of the dentist in identification of systemic conditions contributing to maxillofacial manifestations. This facilitates an early and prompt diagnosis, which in turn affects the treatment and prognosis of the patient.

## Figures and Tables

**Table 1:** Hematological investigations.

Blood parameter	Values (SI unit)	Reference range
RBC	4.29 *∗* 10^12^/L	4.0–5.2 *∗* 10^12^/L
WBC		
Total	**7.0 ** **∗** ** 10** ^9^ **/L**	**4.0–10.0 ** **∗** ** 10** ^9^ **/L**
Differential		
Neutrophils	0.57	0.44–0.68
Lymphocytes	0.32	0.25–0.44
Monocytes	0.66	0.0–0.07
Basophils	0.12	0.0–0.02
Eosinophils	0.175	0.0–0.04
Platelets	2.98 *∗* 10^5^/L	1.5–4.5 *∗* 10^5^/L
Haemoglobin	12.3 g/dL	12.2–18.1 g/dL
ESR	64 mm/hr	8.0–20.0 mm/hr

**Table 2:** Biochemical investigations.

Parameter	Values (SI unit)	Reference range
Serum calcium	8.4 mg/dL	8.5–11.5 mg/dL
Serum creatinine	1.37 mg/dL	0.84–1.4 mg/dL
Total protein	8.75 g/dL	6.6–8.3 g/dL
Serum albumin	3.98 g/dL	3.5–5.2 g/dL
Serum globulin	4.8 g/dL	2.5–4.0 g/dL

**Table 3:** Immunoglobulin assessment.

Parameter	Value (SI)	Reference range
IgG	617 mg/dL	751–1560 mg/dL
IgM	69.3 mg/dL	40.0–230.0 mg/dL
IgA	3000 mg/dL	82.0–453.0 mg/dL
Kappa free light chain	3.7 mg/L	3.3–19.4 mg/L
Lambda free light chain	16.9 mg/L	5.7–26.3 mg/L
Kappa lambda ratio	1 : 5	1 : 1.5
Serum beta-2 microglobulin	2.74 mg/L	0.8–2.4 mg/L

**Table 4:** ISS staging of multiple myeloma.

Stage		Median survival time
Stage 1	Serum beta-2 microglobulin is less than 3.5 (mg/L) and the albumin level is 3.5 (g/dL) or greater	62 months

Stage 2	The beta-2 microglobulin level is between 3.5 and 5.5 (with any albumin level) or The albumin is below 3.5, while the beta-2 microglobulin is less than 3.5	44 months

Stage 3	Serum beta-2 microglobulin is 5.5 or greater.	29 months
